# Improvement in Strain Sensor Stability by Adapting the Metal Contact Layer

**DOI:** 10.3390/s22020630

**Published:** 2022-01-14

**Authors:** Ji-Yeon Choy, Eun-Bee Jo, Chang-Joo Yim, Hae-Kyung Youi, Jung-Hoon Hwang, Jun-Ho Lee, Hyun-Seok Kim

**Affiliations:** Division of Electronics and Electrical Engineering, Dongguk University, Seoul 04620, Korea; jiyeon9711@naver.com (J.-Y.C.); eunbee.j123@gmail.com (E.-B.J.); akreal@naver.com (C.-J.Y.); yeahwooong@gmail.com (H.-K.Y.); junghoonh0326@gmail.com (J.-H.H.); junho_1211@naver.com (J.-H.L.)

**Keywords:** stretchable sensor, strain sensor, wearable device, polyaniline, PDMS

## Abstract

Research on stretchable strain sensors is actively conducted due to increasing interest in wearable devices. However, typical studies have focused on improving the elasticity of the electrode. Therefore, methods of directly connecting wire or attaching conductive tape to materials to detect deformation have been used to evaluate the performance of strain sensors. Polyaniline (PANI), a p-type semiconductive polymer, has been widely used for stretchable electrodes. However, conventional procedures have limitations in determining an appropriate metal for ohmic contact with PANI. Materials that are generally used for connection with PANI form an undesirable metal-semiconductor junction and have significant contact resistance. Hence, they degrade sensor performance. This study secured ohmic contact by adapting Au thin film as the metal contact layer (the MCL), with lower contact resistance and a larger work function than PANI. Additionally, we presented a buffer layer using hard polydimethylsiloxane (PDMS) and structured it into a dumbbell shape to protect the metal from deformation. As a result, we enhanced steadiness and repeatability up to 50% strain by comparing the gauge factors and the relative resistance changes. Consequently, adapting structural methods (the MCL and the dumbbell shape) to a device can result in strain sensors with promising stability, as well as high stretchability.

## 1. Introduction

Highly flexible and stretchable next-generation wearable devices are anticipated for applications in various fields, such as flexible robotics, displays, smart watches for healthcare, and functional clothing. Accordingly, it is appropriate to conduct in-depth research aimed at developing a high-sensitivity strain sensor that can operate with stability despite deformation, while in contact with human skin, in light of the increasing demand for body-mounted devices.

Studies on strain sensors that can convert mechanical strain, such as body movement, into electrical signals have been actively pursued [1]. Novel electrodes and substitutes for typical metal or inorganic films have been studied to ensure conductivity and stretchability under strain, including metal nanowires [2], conductive polymers [3,4], the carbon nanotube (CNT) [5], graphene [6], and composites of these devices.

In particular, polyaniline (PANI), one of the conductive polymers, has attracted attention as an appropriate substance, due to its environmental stability, cost-effectiveness, and tunable conductivity [7], which provides scalability in a multi-functional strain sensor capable of sensing various stimuli, such as chemical gases, temperatures, and humidity [8]. However, strain sensors made by spin-coating or printing PANI dispersed in a solvent are limited because it is difficult to secure high elasticity due to their rigid chain structure.

Cai et al. recently demonstrated stretchability of up to 150% with high sensitivity, and excellent mechanical adaptability via the island-bridge morphology of PANI film using dilute-chemical polymerization, resulting in the expansion of PANI’s versatility as a stretchable electrode [9].

Studies on stretchable electrodes have been actively conducted. However, studies on packaging, integration, and measurement of strain sensors have not been sufficiently conducted [10]. Silver paste for wire connections, conductive tapes such as copper tape, and conductive fabric have been directly attached to stretchable electrodes for estimating their performance [11]. However, such methods have several problems. First, it is difficult to detect minute strain because the deformed region of the stretchable electrode cannot be precisely controlled. Second, contact resistance, based on the unstable attachment and increased instability under external stress, degrades a device’s efficiency. Moreover, the effectiveness of materials that have been used for measurement, including copper and silver, are limited.

There is a restriction in establishing a stable metal semiconductor junction with the various stretchable electrodes. In particular, PANI is a p-type semiconductor whose conductivity results from delocalization by the π-conjugation structure, filling the local electron-deficient part of the cation radical. Thus, its work function is dependent on synthetic methods, such as chemical, electrochemical, and interfacial methods, synthetic conditions, such as the conditions related to dopants, acids, and solvents, and the ambient environment.

The typical work function of PANI is in the range of 4.2–4.7 eV. It should have ohmic contact with metal within this range to achieve high sensitivity [12,13,14]. However, it was not possible to form ohmic contact or to reduce contact resistance by simply employing a metal tape. An additional step, such as using silver paste, has been required [7,9]. In addition, most devices do not come with a separate contact area or appropriate connecting methods [15,16,17]. Therefore, adapting a metal contact layer to sensors could assure ohmic contact.

Silicone-based elastomers, such as polydimethylsiloxane (PDMS), Ecoflex, Dragon Skin, and hydrogel, are commonly used as stretchable substrates for body-attached devices. In particular, PDMS is frequently used due to its high elasticity, chemical stability, biological compatibility, transparency, and thermal stability [18]. Additionally, adjusting the mixing ratio of its components and curing conditions can easily control physical properties [19,20]. However, most studies on PDMS have focused on surface treatment, molding, or adhesion improvement. Hence, fabrication methods for a single substrate that is composed of more than two types of PDMS with different physical rigidities, using their advantages, have not been suggested.

Therefore, only a substrate with a single PDMS is provided for stretchable sensors or their applications. Hence, when a metal thin film that is susceptible to deformation is applied to sensors configured with PDMS, polymers such as polyimide and parylene have been required as a protective layer [21]. Furthermore, although studies on the fabrication of metal patterning on PDMS have advanced, their low surface energy hinders the application of photoresist, due to non-uniformity and low adhesion, preventing precise pattern formation. Additionally, its coefficient of thermal expansion, swelling, and flexibility acts as a hindrance [22,23]. Consequently, the patterning on PDMS via conventional photolithography is difficult and restricted.

This paper introduces a study that ultimately aims to improve the operational stability of strain sensors by adapting the metal contact layer (the MCL). We fabricated a strain sensor that included PANI as a sensing material to detect deformation, the MCL to retain ohmic contact with PANI, and a buffer layer to prevent breakage of the MCL by external stress. Additionally, we cut the sensor in the shape of a dumbbell to maximize the protective effect of the MCL and the buffer layer. Furthermore, the possibility of patterning PANI, the MCL, and the buffer layer is suggested, to provide potential for a diverse selection of materials on stretchable electrodes or the MCL (Au and Ag), indicating the versatility of fabrication of stretchable strain sensors.

The buffer layer formation was established from the facile controllability of PDMS’s physical stiffness. We fabricated a single substrate composed of two different types of PDMS. As a result, it has high elasticity, but partial regions which have low elasticity can be protected from deformation. These partial regions, the buffer layers, can prevent breakage or peel-off of the MCL during the fabrication or application. The buffer layer is PDMS with a Young’s modulus greater than 3 MPa (more than 6 MPa in this study). It is called hard PDMS (H-PDMS) because it is larger than the general Young’s modulus. The substrate that determines the overall elasticity of the sensor has a Young’s modulus less than 1 MPa (less than 700 kPa in this study); it is called soft PDMS (S-PDMS) because its modulus is smaller than the general Young’s modulus. Furthermore, we carried out the PDMS patterning process to formulate PDMS with different physical properties within a single substrate. We introduced the transfer with a sacrificial layer and an auxiliary substrate to minimize damage during the process.

PANI, the MCL, and the H-PDMS can be formed on a single plane with a facile procedure. We demonstrated promising stability, reproducibility, and classification of novel structured sensors based on a comparison of the gauge factors (GFs) of three samples, depending on the MCL and the buffer layer and their transient measurement of relative resistance change under repeated tensile stress. Moreover, current-voltage (I−V) graphs of three types of sensors with different positions of the MCL according to PANI lengths were compared to identify the effect of the dumbbell-shape that was designed in accordance with the standards of the American Society for Testing of Materials (ASTM) [24,25,26].

## 2. Materials and Methods

### 2.1. Materials

Aniline (>99.5%, ACS reagent, 62-53-3), sulfuric acid (98%, for analysis EMSURE, 7664-93-9), hydrochloric acid (37%, ACE reagent, 7647-01-0), (3-Aminoprophyl) triethoxysilane (99%, 2554-06-05), and 2,4,6,8-tetramethyl-2,4,6,8-tetravinylcyclotetrasiloxane (2554-06-05) were purchased from Sigma-Aldrich, Seoul, Korea. A graphite rod (GR002H) was purchased from QRINS, Seoul, Korea. ITO-coated glass was purchased from iTASCO, Seoul, Korea. A silicone elastomer base and a curing agent (Sylgard 184 kit) were purchased from Dow Corning, Midland, MI, USA. VDT-731 (67762-94-1), SIT-6831.2 (68478-92-2), and HMS-301 (68037-59-2) were purchased from Gelest, Morrisville, PA, USA. Copper tape was purchased from 3M, Seoul, Korea. All reagents were used as received without further purification.

### 2.2. Characterization

The thickness of PDMS and PANI was analyzed using a surface profiler (Alpha Step 200, KLA/TENCOR). The alignment of PANI, Au thin film (the MCL), and the H-PDMS were observed using a microscope (MX51, Olympus, Tokyo, Japan). The surface morphology of transferred PANI was analyzed via FE-SEM (S-4800, Hitachi, Tokyo, Japan) at the Korea Advanced Nano Fab Center.

### 2.3. Fabrication of Strain Sensor

#### 2.3.1. PANI/Au/H-PDMS Strain Sensor

**Figure 1:**
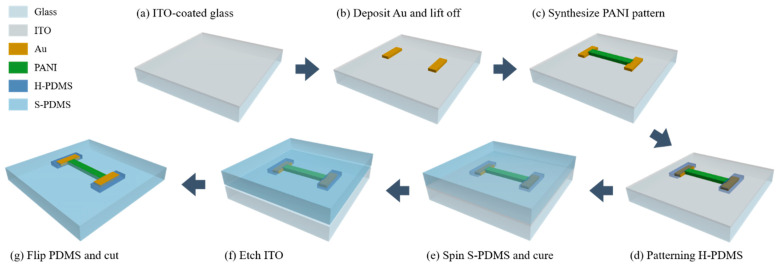
Fabrication of PANI/Au/H-PDMS strain sensor: (**a**) preparing the auxiliary substrate with a sacrificial layer; (**b**) formation of MCL; (**c**) formation of sensing material; (**d**) formulation of the buffer layer; (**e**) formulation of the stretchable substrate; (**f**) separating the stretchable substrate and the auxiliary substrate; (**g**) cutting PDMS into a dumbbell shape.

We introduced a strain sensor with the MCL and the buffer layer (PANI/Au/H-PDMS strain sensor). Its fabrication process is shown in Figure 1. First, we prepared the ITO-coated glass. It was cleaned with acetone, IPA, and deionized water prior to processing. Patterning directly on PDMS may cause alignment difficulties. A transfer process using ITO (185 nm, 10 Ω/cm^2^) as a sacrificial layer and glass as an auxiliary substrate was used (Figure 1a). ITO was chosen as a sacrificial layer because it can act as a working electrode during subsequent PANI synthesis. Typical photolithography was performed to establish the MCL. Photoresist (AZ5214E, AZ Electronics Materials, Charlotte, NC, USA) was spin-coated at 3000 rpm for 30 s. After the exposure, it was immersed in developer (CD-30, AZ Electronics Materials, Charlotte, NC, USA) for 1 min 10 s. Au, which has a significantly larger work function than PANI, was selected as the MCL to form a stable ohmic contact. Thereafter, 150 nm of Au thin film was deposited via an e-beam evaporator (KVE-T5560, Korea Vacuum Tech, Gimpo, Korea), using Au pellets (purity 99.999%) as a source. The deposition conditions were as follows: $2.8\times 10^{-5}$ Torr, 140 mA, and an 0.8 kÅ/s of deposition rate. It was lifted into a rectangular shape (3000 μm×1010 μm) and served as the MCL (Figure 1b).

Next, photolithography was implemented to define PANI capable of detecting mechanical strain. A PANI mask was aligned to overlap with 20 μm of the MCL. A thicker photoresist (KPRO-15, KemLab, Woburn, MA, USA) was used to ensure PANI thickness and spin-coated at 1000 rpm for 45 s. PANI was synthesized electrochemically. Photoresist acted as a deposition mask for patterning (width of 1500 μm and length of  8270 μm). The polymerization conditions were as follows: 0.3 M of aniline and 0.7 M aqueous solution of sulfuric acid as an electrolyte, with the ITO-coated glass as a working electrode and the graphite rod as a counter electrode. The electrodeposition was performed for 1 min 10 s at a fixed voltage of 1.1 V at room temperature [7]. Thereafter, it was baked on the hot plate at 100 °C for 10 min to enhance adhesion to the substrate and dried at room temperature for 24 h to completely remove moisture. It was then soaked in acetone for 10 min to remove the photoresist and the residue was cleaned with IPA and deionized water (Figure 1c).

Subsequently, the H-PDMS patterning was conducted, as described in detail in Section 2.4 (Figure 1d). The S-PDMS determines the stretchability of a strain sensor. It was manufactured by mixing the base and a curing agent (Sylgard 184) with a weight ratio of 15:1. Prior to spin-coating the mixture, the curing agent was spin-coated at 3000 rpm for 30 s to ensure adhesion to the H-PDMS. The mixture was then spin-coated at 500 rpm for 30 s and sufficiently cured at room temperature for 72 h (Figure 1e). Finally, the ITO film was removed by the etchant, which is a diluent of HCl (37%), and deionized water with a weight ratio of 1:3 (Figure 1f). In this step, partial PANI that lost conductivity during the process was oxidized to emeraldine salt form and restored in an acidic etchant having low pH. The PDMS film separated from the glass as the ITO was etched, flipped, and washed with deionized water. After that, it was cut into a dumbbell shape based on ASTM D412 (Figure 1g) [24,25,26]. Additionally, three samples with different PANI lengths (5020 μm,7020 μm, and 8270 μm) were fabricated under the same conditions to optimize the strain sensor according to the position of the MCL and the buffer layer within the same dumbbell structure.

#### 2.3.2. PANI and PANI/Au Strain Sensor

We compared the strain sensor consisting of only PANI (PANI strain sensor) and the PANI/Au/H-PDMS strain sensor to demonstrate the effectiveness of adapting MCL. Additionally, a strain sensor without the buffer layer (PANI/Au strain sensor) and PANI/Au/H-PDMS strain sensor were compared to verify the efficiency of the buffer layer under deformation. The conditions of fabricating PANI, the MCL, and the S-PDMS in these samples were the same as those for the PANI/Au/H-PDMS strain sensors. In the case of the PANI strain sensor, the S-PDMS was applied immediately after defining the PANI on the ITO-coated glass. The sacrificial layer was then etched. PDMS film separated from the glass was cleaned with deionized water and cut into a rectangular shape (7 mm×30 mm). The dumbbell shape was not required because it was composed only of PANI, excluding the MCL and the buffer layer that require structural protection.

The PANI/Au strain sensor was fabricated by coating the S-PDMS on the patterned MCL and PANI. Similarly, the MCL and PANI were aligned with 20 μm of the overlapping area. The surface treatment with (3-Aminoprophyl) triethoxysilane (APTES) was processed to enhance the adhesion between the S-PDMS and the MCL just before the S-PDMS coating. An aqueous solution diluted APTES with a weight ratio of 1:100 was used. It was dried with an N_2_ gun without a further washing process after immersion of 20 min [27]. After that, the S-PDMS was coated and cured for a sufficient time. The sacrificial layer was removed using HCl diluent to isolate it from the glass. It was washed by deionized water and cut into the dumbbell shape to protect the MCL structurally.

### 2.4. Patterning the H-PDMS

**Figure 2:**
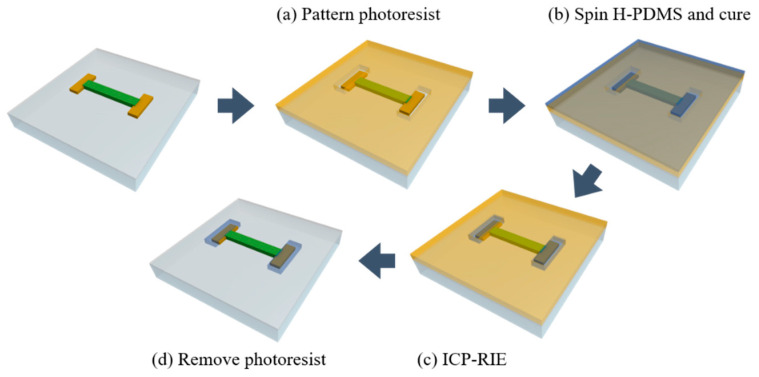
The process of patterning the H-PDMS: (**a**) photoresist as an etching mask; (**b**) formation of H-PDMS; (**c**) etching the top of H-PDMS to expose photoresist surface; (**d**) remained H-PDMS as the buffer layer.

We applied the H-PDMS with a high Young’s modulus as the buffer layer to protect the MCL from deformation. We introduced its patterning process as shown in Figure 2. First, the thick photoresist was applied to the sample to complete the step shown in Figure 1c, to ensure sufficient thickness that is fundamental for a durable buffer layer. We spin-coated the photoresist (KPRO-15, KemLab, Woburn, MA, USA) at 1000 rpm for 45 s and soft-baked it to form the first photoresist layer. After cooling, a sufficient thickness of 35~40 μm was obtained by repeating the same process under identical conditions. Thereafter, a pattern with a size of 4000 μm×1500 μm was aligned with PANI overlapped 10 μm and the bulk MCL via conventional photolithography (Figure 2a). To develop the photoresist to a thickness greater than 35 μm, it was immersed in developer (CD-30, AZ Electronics Materials, Charlotte, NC, USA) for 20 min. APTES surface treatment was performed prior to applying the H-PDMS to improve adhesion between the MCL and the H-PDMS. After immersing in APTES aqueous solution diluted at a weight ratio of 1:100 for 20 min, it was taken out and dried with an N_2_ gun.

The H-PDMS was established in the following steps. First, 3.7 g of VDT-731 and 50 μL  of Pt catalyst (SIP-6831.2) were mixed. Then, 0.1 g of modulator (2,4,6,8-tetramethyl-2,4,6,8-tetravinylcyclotetrasiloxane) and 1 g of HMS-301 were added and mixed. The mixture was degassed whenever each substance was added [28,29]. The mixture was spin-coated at 1000 rpm for 30 s. Self-leveling for 10 min on a flat place at room temperature was performed immediately after the preceding step. It was then cured in an oven at 60 °C for 4 h (Figure 2b). ICP-RIE was performed to expose the top surface of the photoresist by etching the H-PDMS. The selectivity of the H-PDMS photoresist was 1.5:1. The following were the ICP-RIE conditions: 500 W of RF power, 200 W of RF bias power, 10 mTorr of pressure with 90 sccm of SF_6_ and 6 sccm of O_2_, which lasted 32 min (Figure 2c) [30]. It was then immersed in an acetone solution to remove the photoresist. Notably, the exposed top surface facilitated its removal. The thickness of the remaining H-PDMS was 12 μm. The H-PDMS containing 10 μm of PANI and the bulk MCL and an overlapped area with 10 μm of the MCL and bulk PANI, situated externally to the H-PDMS, guaranteed the interconnection of each part under strain. Additionally, we manufactured Ag/H-PDMS samples and observed the variation of the surface conditions and the resistance based on strain to verify the effectiveness of the H-PDMS. Ag (150 nm) as the MCL and the H-PDMS (25 μm) as the buffer layer were progressed under the same conditions as mentioned above. The S-PDMS was also formed identically using the mixture of a Sylgard 184 kit with a 15:1 weight ratio, and cut into the dumbbell shape.

### 2.5. Measurement Conditions

**Figure 3:**
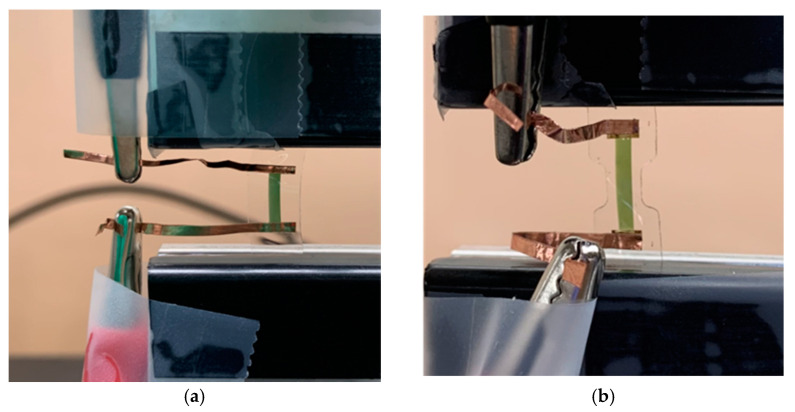
Measuring electrical properties. (**a**) Sample A (PANI strain sensor), (**b**) Sample B (PANI/Au strain sensor), and Sample C (PANI/Au/H-PDMS strain sensor).

We prepared three different types of samples with and without the Au thin film as the MCL and the H-PDMS as the buffer layer: Sample A (PANI strain sensor), Sample B (PANI/Au strain sensor), and Sample C (PANI/Au/H-PDMS strain sensor). Sample A was cut into a rectangular shape because it lacked metallic thin film and was vulnerable to external stress. Samples B and C were cut into the shape of the dumbbell based on the ASTM D412 model to maximize the efficiency of the MCL protection. Each sample had a PANI pattern, 1500 μm of width and 8270 μm of length, which can detect mechanical deformation. We also prepared three different lengths (5020, 7020, and 8270 μm) of PANI in Sample C to explore the effect of the dumbbell structure. The copper tape was prepared in size 0.125 cm×3 cm and attached directly to PANI or the MCL to measure the electrical properties (Figure 3). The measurement was repeated consecutively for all samples under the same conditions to obtain I−V characteristics. Measurement conditions were as follows: dual sweep from −10 V to 10 V with 0.1 V step voltage in a fixed state with deformation at 0%, 10%, 20%, 30%, 40%, and 50%. Transient characteristics were measured at speeds of 50 mm/min and 6 mm/min. The former had a waiting time of 10 s, and the latter had a waiting time of 0.1 s. Ag/H-PDMS samples were cut into the ASTM D412 model, and their surface condition was observed using a microscope at 0%, 20%, and 60% strains. Resistance was measured under the 0%, 20%, 40%, and 60% strains using the Au probe.

## 3. Results

### 3.1. PANI Morphology

**Figure 4:**
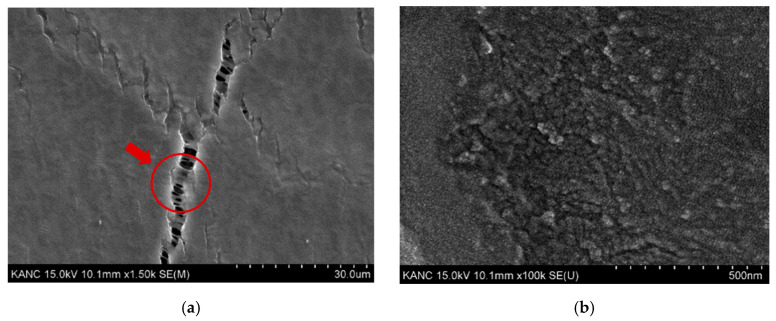
SEM image of transferred PANI film: (**a**) the island-bridge structure; (**b**) enlarged image of PANI film.

It was important to maintain the interconnection of PANI under high tension to have reversible tensile strength. Therefore, the construction of an island-bridge structure was significant because the previous study showed its effectiveness of up to 150% [9]. In the earlier study, we found that electrochemically synthesized PANI formed the initial thin film that served as an island, and the nanowires that grew on it acted as a bridge. Figure 4a shows the scanning electron microscope (SEM) image of PANI taken after transfer with PDMS, depicting island-bridge constitutions. Figure 4b is the enlarged image, revealing the organization with small dots instead of nanowires. These small dots, the reverse side of the PANI film, appeared because the transfer process caused PDMS to embed it. Therefore, the nanowires of the island-bridge buried in this way were able to tolerate up to 50% strain.

### 3.2. The MCL Protection Effect of the Dumbbell Structure and the H-PDMS

**Figure 5:**
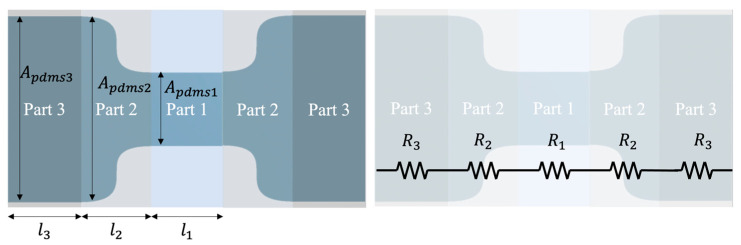
An equivalent circuit model of dumbbell shape.

Figure 5 is an equivalent circuit model of the dumbbell shape of Sample C. Part 1 is the narrowest area, Part 2 is the curved area, and Part 3 is the widest area. The resistance of PANI formed in each area is denoted by $R_{1}$, $R_{2}$, $R_{3}$. The equation for the resistance of PANI ($R_{pani}$) is expressed in Equation (1), and a Young’s modulus of the S-PDMS (E) is presented in Equation (2). ρ is the resistivity of PANI, l is the length of PANI in the corresponding part ($l_{1}\;$= 2.4 mm, $l_{2}\;$= 1.6 mm, $l_{3}\;$= 1.335 mm), and $A_{pani}$ is the cross-sectional area of PANI. Since E is related to the S-PDMS, F is the force applied to the S-PDMS, and $A_{pdms}$ is the cross-sectional area of the S-PDMS ($A_{pdms1}\;$= 3t mm, $A_{pdms2}\approx \;$4.6t mm, $A_{pdms3\;}$= 6.26t mm, t = thickness of the S-PDMS).

(1) $$R_{pani}=\frac{\rho l}{A_{pani}}$$

(2)  E=stressstrain=FApdmsΔll

The strain (S) of Part 1 can be expressed as $S_{1}$ (Equation (3)). Then, the changes in S, when $A_{pdms}$ are different, can be expressed in Equations (4) and (5).

(3) Δl1l1=S1

(4) $$S_{2}=S_{1}\frac{A_{pdms1}}{A_{pdms2}}$$

(5) $$S_{3}=S_{1}\frac{A_{pdms1}}{A_{pdms3}}$$

The change in resistance ($\Delta R_{pani}$) can be obtained based on the change in PANI’s length (Δl) (Equations (6)–(8)). By referring to Equations (3)–(5), Δl can be expressed by the equations related to $S_{1}$ (Equations (9) and (10)). As a result, the dumbbell structure is protective of the MCL because $\Delta l_{3}$ is the smallest.

(6) $$\Delta R_{1}=\frac{\rho \Delta l_{1}}{A_{pani}}$$

(7) $$\Delta R_{2}=\frac{\rho \Delta l_{2}}{A_{pani}}$$

(8) $$\Delta R_{3}=\frac{\rho \Delta l_{3}}{A_{pani}}$$

(9) $$\Delta l_{2}=l_{2}S_{2}=l_{2}S_{1}\frac{A_{1}}{A_{2}}$$

(10) $$\Delta l_{3}=l_{3}S_{3}=l_{3}S_{1}\frac{A_{1}}{A_{3}}$$

**Figure 6:**
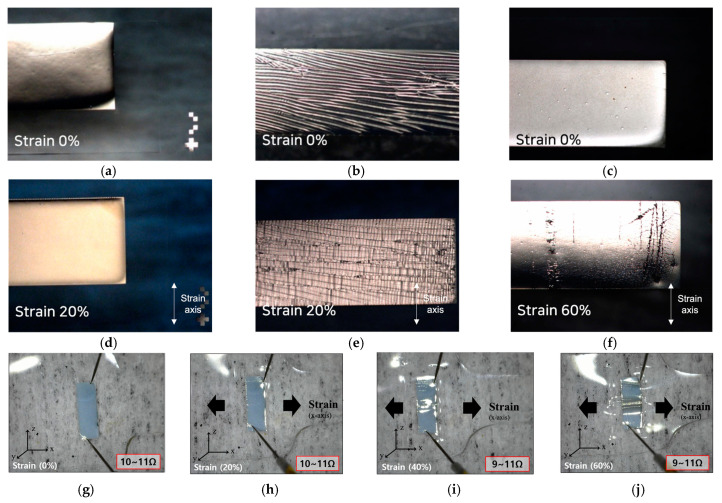
Ag/H-PDMS and Ag-only samples: (**a**) 0% of the Ag/H-PDMS sample; (**b**) 0% of the Ag sample; (**c**) 0% of the Ag/H-PDMS; (**d**) 20% of the Ag/H-PDMS sample; (**e**) 20% of the Ag sample; (**f**) 60% of the Ag/H-PDMS sample; (**g**–**j**) overall image of the Ag/H-PDMS sample and the measured resistance at (**g**) 0%, (**h**) 20%, (**i**) 40%, and (**j**) 60%.

We compared the Ag/H-PDMS and Ag-only samples to verify the efficiency of the buffer layer and the dumbbell shape. We also observed the surface and the resistance according to strain. The strain-dependent surface changes of the Ag thin film in the Ag/H-PDMS and the Ag samples are shown in Figure 6a–f. At the initial state (0%), the Ag/H-PDMS samples completely protected the surface (Figure 6a,c). However, the Ag samples immediately adhered to the S-PDMS and damaged the surface even in the absence of applying artificial stress (Figure 6b). In particular, the Ag/H-PDMS samples’ initial condition was maintained even at 20% strain (Figure 6d). On the contrary, the Ag samples showed cracks both coincident with and perpendicular to the strain axis (Figure 6e).

Figure 6g–j shows the images of one side of the dumbbell shape, its Ag thin film, and the H-PDMS in the Ag/H-PDMS samples. Figure 6g,h shows that Ag thin film keeps its outline under deformation, under strain of up to 20%. However, it also shows distortion in the *z*-axis at 40% and 60% strains, causing destruction in the same direction as the strain axis. As shown in Figure 6f, the Ag/H-PDMS samples experienced damage that was parallel to the strain axis under high tension.

Although the H-PDMS had sufficient rigidity to protect the Ag films from strain, the difference in elasticity between the S-PDMS and the H-PDMS, and their high flexibility, caused these cracks (Figure 6i,j). When stress was applied in the x-axis, the S-PDMS, having high elasticity, stretched in the same axis as the stress applied, and decreased in width in the y-axis at the same time. However, the S-PDMS beneath the H-PDMS could not be stretched due to the H-PDMS being at the top, with its low stretchability. Therefore, the H-PDMS-formed regions were bent in the z-axis to compensate for the width. This implies that in the case of samples with the buffer layer, the parallel detriments appeared due to flexibility and not due to stretching.

The resistance measurement was carried out to evaluate the performance. The probes were positioned from edge to edge of the Ag thin film to maximize the effect of its breakage caused by deformation. Despite this damage, all the results measured at strains from 0% to 60%, with a 20% interval, had 9–11 Ω, indicating stability.

### 3.3. I−V Measurement

**Figure 7:**
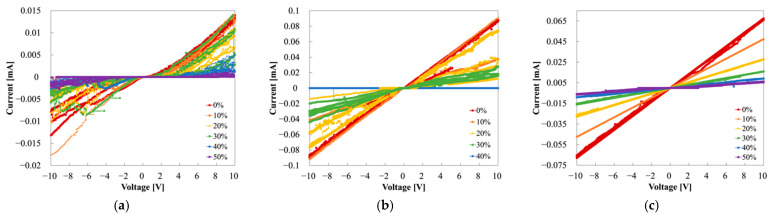
I−V measurement of Samples A–C: (**a**) Sample A (PANI strain sensor), (**b**) Sample B (PANI/Au strain sensor), and (**c**) Sample C (PANI/Au/H-PDMS strain sensor).

**Figure 8:**
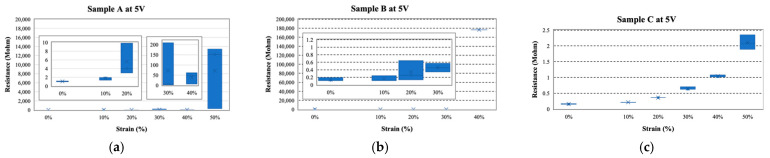
Resistance vs. strain of Sample A−C: (**a**) Sample A (Insets show magnified view due to large resistance differences), (**b**) Sample B (the inset shows a magnified view), and (**c**) Sample C showing distinguishable classification of the strain.

We measured the I−V characteristics of Samples A, B, and C to evaluate the stability and elasticity of the MCL-adapted and the buffer layer-adapted strain sensor. Figure 7 shows the I−V measurements of each sample at strains of 0%, 10%, 20%, 30%, 40%, and 50%. Through the I−V curves, the linearity and reproducibility of each sample were exhibited. GF, a parameter indicating sensitivity, verified the ohmic contact of PANI and the MCL, and their stability. GF was calculated as the change in relative resistance (Δ*R*) with strain (*ε*) (Equation (11)). The relative resistance change (Δ*R*) is described in Equation (12), where *R*_0_ denotes the resistance in the initial state (0%) and *R* represents the resistance in the strain state. For the estimation, each resistance was described in the following colors: red for 0%; orange for 10%; yellow for 20%; green for 30%; blue for 40%; and purple for 50%. Figure 7a shows an exponential aspect and randomly overlapped values of Sample A. At the 30%, 40%, and 50% strains, the currents were decreased, with increasing strain. However, the result was almost the same at 0%, 10%, and 20%, regardless of the strain. Instability was indicated with disparate results, despite repeated measurements under the same conditions. At 30%, the first measurements had a value of approximately 0.015 mA (biased at 10 V). However, the second measurement had a value of 0.005 mA, showing an error of 67% even with the same estimation conditions. Additionally, the current at 10% strain was similar to that at 0% strain at a positive voltage bias, but the current was similar at 30% and 40% in the case of a negative voltage, rather than 0%. This asymmetry implied that PANI in Sample A, with copper tape, had unstable contact. The I−V curves of Samples B and C showed more linear curves than did Sample A, as shown in Figure 7, because the MCL formed ohmic contact with PANI. However, Sample B was insulated at 40% strain and also emitted some noises. Figure 8 is the plot of resistance vs. strain when 5 V is applied. The resistance was measured consecutively three times at each strain. The elasticity of the strain sensor damaged the MCL, which caused noises under the strain during the measurements and had a fatal effect on strain determination. The linearity or tendency of resistance according to strain was not observed in Samples A and B (Figure 8a,b). In contrast, Sample C showed clear I−V curves and resistance according to each strain (Figure 7c and Figure 8c). By employing the MCL and the H-PDMS, noises could be suppressed and could improve the sensor’s reliability and stability.

(11) $$\mathrm{GF}=\frac{\Delta R}{\varepsilon }$$

(12) $$\Delta R=\frac{R-R_{0}}{R_{0}}$$

**Figure 9:**
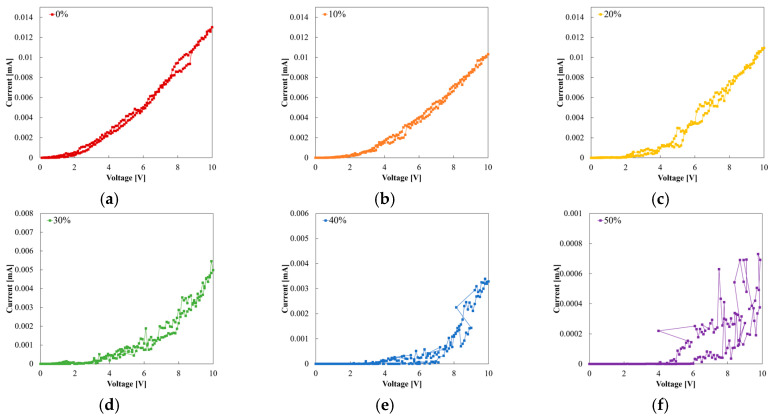
Individual I−V curves of Sample A based on strain: (**a**) 0% strain, (**b**) 10% strain, (**c**) 20% strain, (**d**) 30% strain, (**e**) 40% strain, and (**f**) 50% strain.

Figure 9 shows the individual graphs in each strain of Sample A. Figure 9a–f show that Sample A has strain-dependent threshold voltages: 2 V at 0% and 10% strain; 2–4 V at 20% and 30% strain; and 4–6 V at 40% and 50% strain. These values indicate that the threshold voltage increases with increasing strain. Figure 9 also shows more sporadic data distribution and hysteresis with increasing strain. Hence, we observed that the current at 10 V of each strain generally decreased with rises in strain. However, currents at 20% strain had exceptional results, having larger values than 10%. This irregularity can be interpreted according to the work function and contact resistance of copper tape and PANI, as mentioned above.

**Figure 10:**
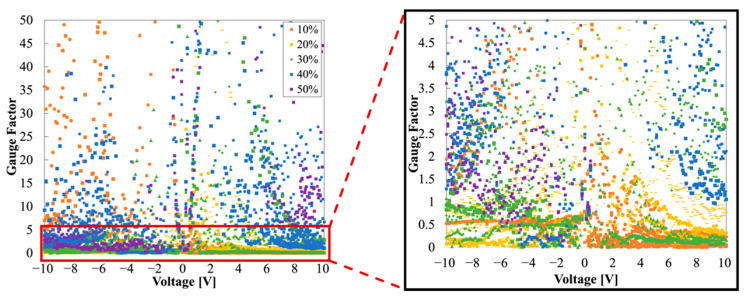
Overall GF of Sample A (**left**) and enlarged GF of Sample A (**right**).

GFs of Sample A are shown in Figure 10. Considering GFs at 10%, they were presented between 0 and 0.5 when positive voltage-biased, whereas they were around 0.5 at the negative voltages, showing an asymmetric distribution. Similarly, GFs were distributed between 0 and 0.5 at the positive voltages at 30%; however, GFs were between 0.5 and 1 at the negative voltages. Notably, it was impossible to discriminate between the strain based on the GFs in these two cases because the GFs at the positive voltages were overlapped in the same range of 0–0.5. At 20% and 40%, GFs of both sides of the voltages showed a relatively symmetrical tendency. In contrast, GFs of 50% had asymmetry, with GFs of 10 or more at the positive side and 0–5 at the negative side. The unstable behavior in Sample A was illustrated through the GFs’ distribution. The asymmetry of the GFs is the result of the Schottky junction of PANI and the copper tape.

**Figure 11:**
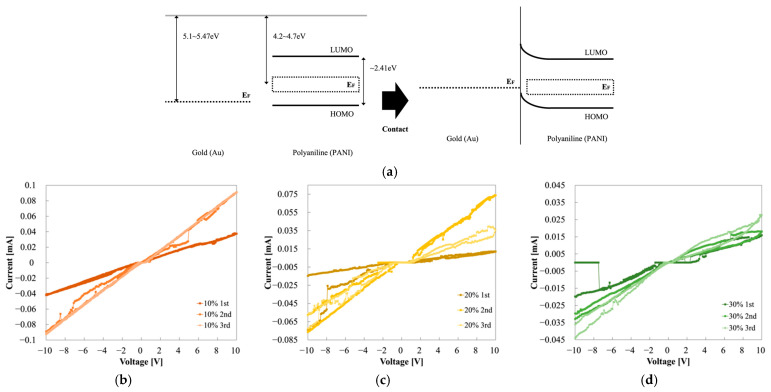
(**a**) Energy band diagram of PANI and the MCL before contact (**left**) and after contact (**right**); the individual I−V curves according to strain in Sample B: (**b**) 10% of strain, (**c**) 20% of strain, and (**d**) 30% of strain.

The individual I−V graphs for each strain of Sample B are shown in Figure 11b–d. Each graph is the result of three consecutive measurements under the same conditions. Sample B used Au as the MCL, which has a larger work function than PANI, and a stable ohmic contact can be formed. Thus, Sample B was significantly more linear than Sample A at 0% strain. However, Sample B showed undesirable behaviors, such as large fluctuations in the I−V curves through multiple measurements when the strain was applied as shown in Figure 11b,c. The I−V curves for 10% strain shown in Figure 11b indicate that the first measurement has the lowest value, and the second and third measurements have increased. The MCL was damaged as the strain applied, and the resistance rose rapidly and exhibited its lowest value in the first trial. It partially recovered and stabilized due to the elasticity of the S-PDMS. The resistance caused the elevated currents in the second and third trials. Similarly, the graph for 20% in Figure 11c depicts that the first trial had the lowest value and current increased in the second trial and decreased in the third. As with Figure 11b, Figure 11c shows a drastic and immediate rise in the resistance at the moment when stress was applied. The damaged MCL recovered with time, leading to the current increase at the second measurement. The third measurement was lower than the second one in contrast to the interpretation described at 10% strain. This might be explained by the fact that the MCL was damaged when the strain was applied, even though the dumbbell structure and the elasticity of the S-PDMS protected the MCL. Therefore, the resistance increased, negatively affecting the sensor’s behavior under the strain. At 30%, the I−V curves had the lowest value at the first trial, increasing at the second and third trials (Figure 11d). The range of current fluctuations according to the number of trials was small, unlike the previous range at 10% and 20%, which was different from the other two cases where the damage to the MCL was noticeably recovered by the stretchability of the substrate. At 30%, the MCL could not revive its properties, or it may have needed sufficient time to recuperate because of the large stress and deformation. Therefore, the MCL with the H-PDMS would be truly necessary.

**Figure 12:**
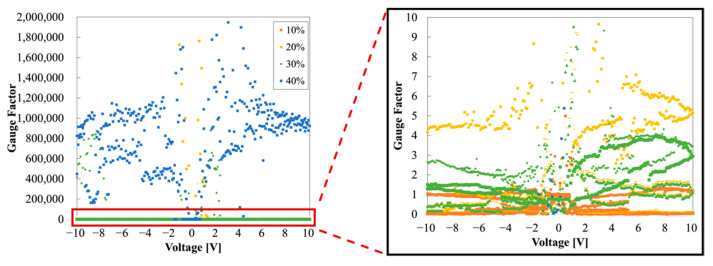
Overall GF of Sample B (**left**) and enlarged GF of Sample B (**right**).

The GFs of Sample B are shown in Figure 12. GFs at 40% were over 1,000,000 because of the MCL’s destruction. GFs at 10%, 20%, and 30% were spread between 0 and 10 and made it difficult to discriminate among the strains, owing to their wide distributions. Additionally, fluctuation in hysteresis and the unsettled results following the damaged MCL negatively affected the sensor performance.

**Figure 13:**
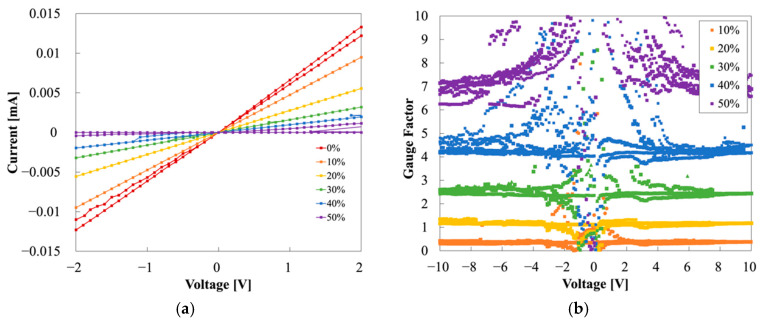
(**a**) I−V characteristics of Sample C with a voltage range -2 V–2 V; (**b**) GFs of Sample C.

As shown in Figure 7c, Sample C has clearly differentiated I−V measurements for each strain, as the most linear, stable, and repeatable. Specifically, the strain was linear and distinguishable even at the low voltages of −2 V–2 V, as shown in Figure 13a. Furthermore, when comparing the currents at 2 V in the initial state of Samples A and C, the current in the latter was 48 times larger than in the former. The MCL and PANI connection had smaller contact resistance than the copper tape and PANI connection. The GFs of Sample C are shown in Figure 13b: GFs of 0.5 at 10%, GFs of 1 at 20%, GFs of 2.5 at 30%, GFs of 4–5 at 40%, and GFs of more than 6 at 50%. The GFs were completely differentiated and characterized according to each strain. Moreover, the ohmic contact between PANI and the MCL was verified via the symmetric GFs distribution through the positive and negative sides. In general, a high GF indicates high sensitivity of the strain sensor, but at the same time, high-precision and expensive equipment is required to detect large resistance (>10^9^ Ω) [7]. Table 1 shows the gauge factors, depending on the measurement contact method. Therefore, Sample C, with a relatively small but highly distinguishable GF will be promising for further body-mounted wearable device applications.

**Figure 14:**
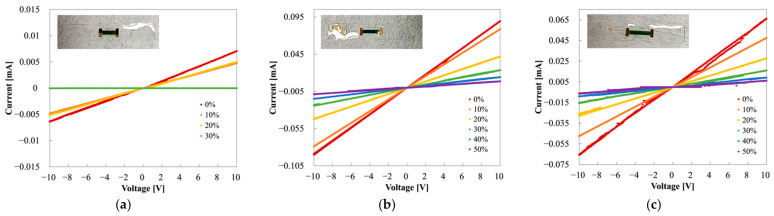
I−V curves of the PANI/Au/H-PDMS strain sensor according to the different lengths of PANI: (**a**) 5020 μm, (**b**) 7020 μm, and (**c**) 8270 μm.

Figure 14 shows the I−V curves of PANI/Au/H-PDMS strain sensors with three different PANI lengths. Each inset shows a sensor with different PANI lengths: 5020 μm, 7020 μm, and 8270 μm, respectively. The I−V measurement of the strain sensor with the PANI length of 5020 μm is shown in Figure 14a. We observed the insulation due to the rapid rise of resistance under stress with increasing strain from 20% to 30%. When the length of PANI shortened, the MCL and H-PDMS were positioned on the curved area of the dumbbell, no longer protected by the structure. The I−V measurements of the strain sensors with PANI lengths of 7020 μm and 8270 μm are shown in Figure 14b,c, respectively. They had distinctly isolated values according to deformation and sustained conductivity, even at 50% strain. Both samples, the MCL and the H-PDMS, placed on a wide area of the dumbbell, were structurally sufficient to protect it from the strain, resulting in stable outcomes.

### 3.4. Transient Measurement

**Figure 15:**
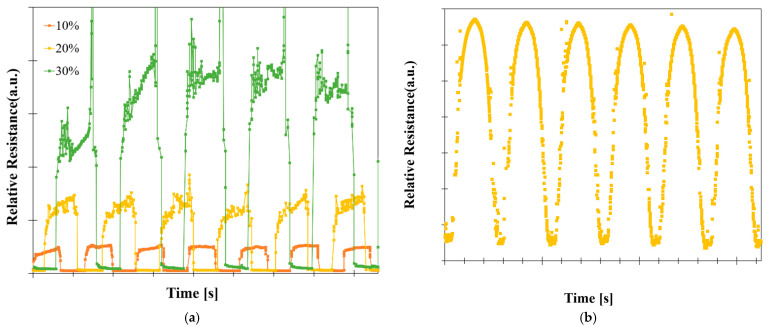
Transient measurement of Sample C: (**a**) relative resistance change as a function of time at 10%, 20%, and 30% strain; (**b**) transient curve at the tensile speed of 6 mm/min and a waiting time of 0.1 s.

Figure 15a shows the transient measurement of Sample C, which was pulled and recovered at the speed of 50 mm/min and 10 s of waiting time. It shows the relative resistance changes with time, at 10%, 20%, and 30% strain. The relative resistance change at 20% strain is two times greater than that of 10%, and that at 30% is four times greater than that of 20%. This indicates that the relative resistance change can be a parameter for classifying deformation. In particular, intense overshooting occurred with increasing strain due to the rapid and instantaneous changes in the relative resistance caused by the sudden increase in resistance at the moment of pulling. Figure 15b, which was pulled and recovered at the speed of 6 mm/min, indicates a gradual relative resistance change without the overshooting phenomenon. Consequently, it demonstrates that relative resistance change can be a parameter for classifying deformation but can be affected by tensile speed.

## 4. Conclusions

We introduced the strain sensor to the adapting MCL to ensure ohmic contact with PANI and formed a buffer layer to protect it from the strain. The I−V curves according to each strain showed that the sample containing only PANI had the most unstable behavior and suggested that PANI and copper tape had Schottky contact, prolonging performance degradation. The strain sensor with the MCL, but without the H-PDMS, had deterioration due to damage to the MCL under deformation. The PANI/Au/H-PDMS strain sensor had stable behavior that helped in distinguishing the characterization and classification. As a result, we demonstrated the improvement of operational stability by adapting the MCL and the buffer layer. Moreover, this arrangement is more suitable for body-mounted wearable devices because it is stable even at a low voltage. The fabrication of the substrate with different stretchability regions on a single plane provided a research foundation for strain sensors’ integration. We anticipate various future research into structural enhancement, such as the measurement or packaging of a strain sensor, rather than the development of materials, which can improve a device’s performance and sustainability, to contribute to the commercialization of stretchable devices.

## Figures and Tables

**Table 1:** The comparison of the measurement contact method of PANI-based strain sensors.

Measurement Contact Method	Gauge Factor	Materials	Ref.
Copper tape and silver glue	54 at 50% strain	PANI	[7]
Copper tape and silver paste	140 at 100% strain	PANI	[9]
Silver fabric	74 at 1% strain	PANI	[31]
Copper wire and silver paste	6.725 at 0~120% strain	PANI/TPU	[32]
MCL	6 at 50%	PANI	This work
